# Begomoviruses associated with okra yellow vein mosaic disease (OYVMD): diversity, transmission mechanism, and management strategies

**DOI:** 10.1186/s43897-024-00112-4

**Published:** 2024-11-05

**Authors:** Thomas Wilbur Davis, Andrew Nasa Thompson

**Affiliations:** 1 Doctor of Plant Health, University of Nebraska – Lincoln, Lincoln, NE 68508 United States; 2 Resource Utilization and Plant Protection, China Agricultural University, 17 Qinghua Donglu, Beijing, 100083 China

**Keywords:** Whitefly (*Bemesia tabaci*), Okra Yellow Vein Mosaic Virus (OYVMV), Transmission mechanism

## Abstract

Okra yellow vein mosaic disease (OYVMD) is a major constraint to okra production globally. It is caused by several distinct *begomoviruses*, including okra yellow vein mosaic virus (OYVMV), that are transmitted by the whitefly. This study synthesizes current knowledge on the complex interactions between whiteflies, *begomoviruses*, and okra plants that enable viral spread and cause OYVMD. The acquisition and transmission cycle involves specific processes including virion ingestion during phloem-feeding, endocytosis and passage across insect tissues, secretion in saliva, and inoculation into plants. Molecular compatibilities between vector coat proteins, midgut proteins, and plant factors modulate virus replication and movement through barrier tissues. Abiotic stresses and host traits also impact whitefly behavior and virus epidemiology. *Begomoviruses* such as OYVMV have spread globally wherever whitefly vectors and susceptible okra varieties occur. Integrated management of the tripartite pathosystem that incorporates host resistance, cultural tactics, and biological control is required to mitigate the transmission of *begomoviruses* and OYVMD impact. Finally, resolving vector-virus interactions and developing interference strategies will help contribute to strengthening okra germplasm resistance which can support sustainable food production.

## Introduction

Plant viruses, including *begomoviruses*, pose significant challenges to crop production worldwide. *Begomoviruses* are a major threat to okra (*Abelmoschus esculentus*) cultivation, as the disease Okra yellow vein mosaic disease (OYVMD), caused by many species of this virus, such as Okra yellow vein mosaic virus (OYVMV) (GenBank accession number NC_004673.1) and Okra enation leaf curl virus (OELCuV), (GenBank accession number KC019308) is particularly detrimental to okra production (Davis 2022a; Hameed et al. 2014; Naresh et al. 2019). These viruses are transmitted by insect vectors, primarily whitefly *Bemisia tabaci* (Hemiptera: Aleyrodidae). Over the years, *begomoviruses* have spread to many okra-growing regions in Asia, Africa, and South America (Bragard et al. 2013). *Begomoviruses* are transmitted in a persistent circulative manner exclusively by *B. tabaci* (Bragard et al. 2013; Kumar et al. 2018) and are all notable causes of OYVMD. OYVMD was first reported in India in 1924 (Kulkarni 1924), and has since spread to okra-growing regions worldwide. The disease is characterized by yellow vein clearing, vein chlorosis, leaf curling, and overall stunting. Several *begomoviruses* have been associated with OYVMD in different countries, solidifying the complexity of this disease (Venkataravanappa et al. 2013b).

**Fig. 1:**
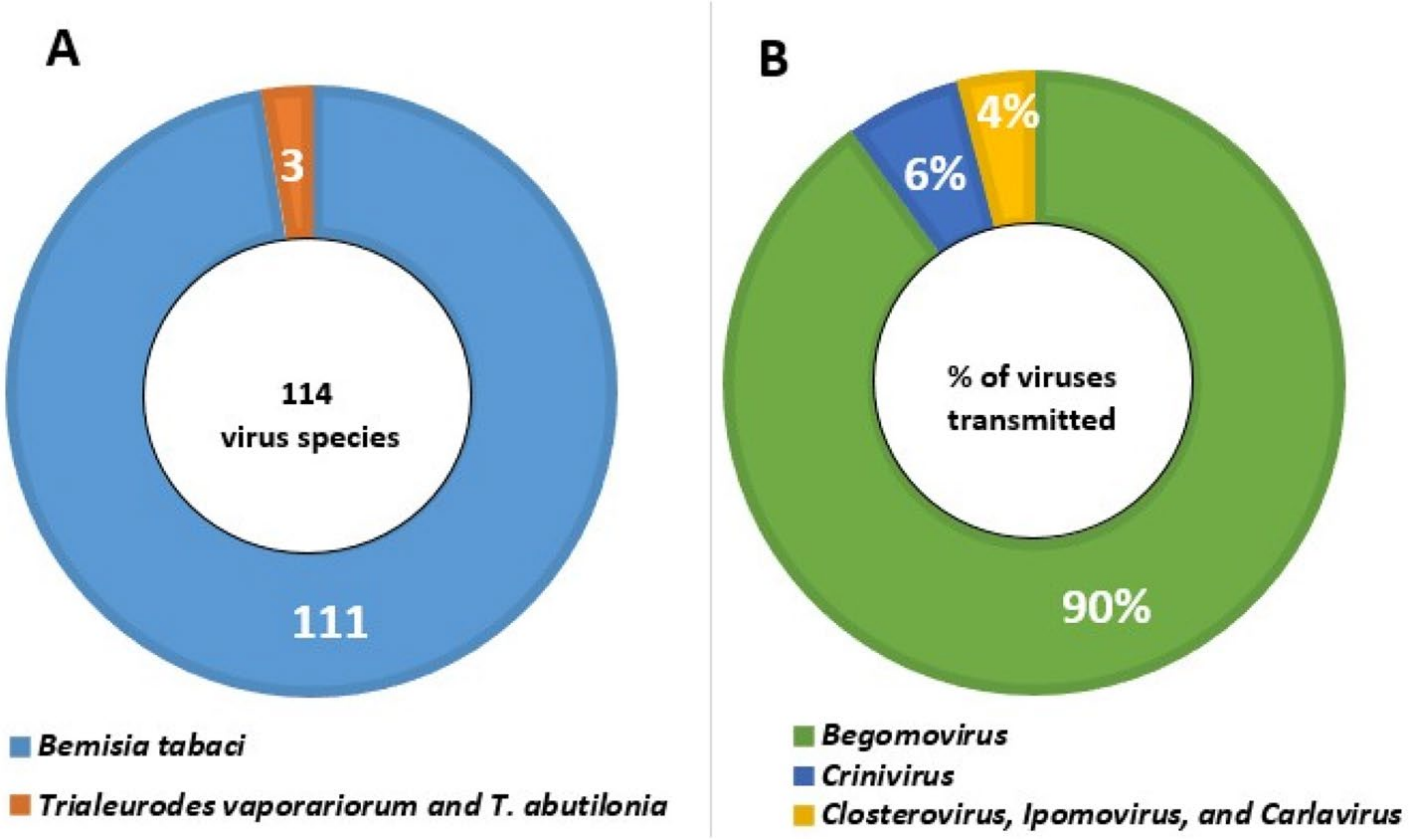
Species of whiteflies in the Aleyrodidae family that transmit plant viruses (**A**), and the viruses they transmit (**B**)

*B. tabaci* is an invasive pest from the family Aleyrodidae that is a major limiting factor to agricultural productivity worldwide owing to its ability to transmit a wide range of plant viruses. *B. tabaci* is a vector for numerous plant viruses belonging to the genera *Begomovirus*,* Carlavirus*,* Ipomovirus*, and *Closterovirus* (Navas-Castillo et al. 2011). In general, the Aleyrodidae family of whiteflies is responsible for the transmission of 114 different virus species. Of them, 111 are transmitted by *B. tabaci*, and three others by *Trialeurodes vaporariorum* and *T. abutilonia*. The *Begomovirus* genus accounts for 90% of the virus species spread by whiteflies, followed by the *Crinivirus* genus at 6%, the *Closterovirus*,* Ipomovirus*, and *Carlavirus* genera at 4% (Fig. 1). As of yet, other viruses that are named and spread by whiteflies are not classified as species (Jones 2003).

*B. tabaci* is among the most devastating pests of vegetable and fiber crops globally. Okra yellow vein mosaic disease, caused by *begomoviruses* transmitted by *B. tabaci*, poses a significant threat to okra production. Understanding the molecular mechanisms of the virus-vector interaction and the resulting disease is critical for developing novel control strategies against the viruses and their vectors (Liu et al. 2013). While the retention sites of *begomoviruses* in the vector’s body have been identified, the molecular interactions enabling circulation and transmission are not fully understood - making further research on *begomovirus*-vector interactions during the circulative transmission process needed. The objectives of this paper are to summarize current knowledge on the transmission mechanisms of *begomoviruses* causing OYVMD by the vector *B. tabaci*, identify gaps in understanding *begomovirus*- *B. tabaci* interactions, and discuss future research directions that can provide insights into interrupting *begomovirus* transmission. Understanding the molecular basis of circulative transmission by the *B. tabaci* vector will support the development of novel genetic resistance strategies in the okra host and transmission-blocking methods for *begomovirus* and OYVMD management.

## *B. Tabaci*: vector of *Begomovirus*

There are 1556 whitefly species documented globally (Sani et al. 2020). Among these, *B. tabaci* stands out as a destructive species, inflicting significant economic damage on vegetable and ornamental crops across various agricultural regions. *B. tabaci* is one of the most widespread and prolific crop pests globally. It has a broad host range, feeding on over 900 plant species (De Barro et al. 2011). This insect that eats many types of plants harms them directly by feeding on them and indirectly by spreading over 100 plant viruses, such as the *begomovirus* (Navas-Castillo et al. 2011). The *B. tabaci* is a cryptic species complex that is made up of at least 44 species that look the same but differ in biological traits and genetics (De Barro et al. 2011; Dinsdale et al. 2010). The most predominant and damaging of these are Middle East-Asia Minor 1 (MEAM1, formerly biotype B) and the Mediterranean (MED, formerly biotype Q) (De Barro et al. 2011). Both MEAM1 and MED have spread globally in the past 30 years, often displacing indigenous whitefly populations, and causing major agricultural losses (Brown et al. 1995; Perring 2001). MEAM1 was originally described from Israel, Jordan, Pakistan, and India in the 1980s. It has a pan-tropical distribution and is regarded as one of the most invasive and destructive whiteflies globally. MEAM1 is a major pest of food and fiber crops with high levels of resistance to insecticides (De Barro et al. 2011; Dinsdale et al. 2010). MED, on the other hand, is native to the countries surrounding the Mediterranean Sea but now similarly spread globally. It is an important pest of greenhouse crops worldwide that exhibits high levels of resistance to neonicotinoid insecticides (Gorman et al. 2010; Li et al. 2021). Other notable species are Asia-1, *B. tabaci* New World, and *B. tabaci* China 1. The Asia-1 species of *B. tabaci* is an indigenous species that is dominant in Thailand. It is not clear where exactly the Asia-1 species of *B. tabaci* originates from (Kanakala and Ghanim 2019). The *B. tabaci* New World is indigenous to the Americas but has now spread to other regions and regarded as a minor pest species compared to MEAM1 and MED, with limited resistance to insecticides reported (De Barro et al. 2011; Ghosh et al. 2015). The *B. tabaci* China 1 was first detected in China in the 1990s and now prevalent across Asia. It is an important pest of vegetables and ornamental crops in China, with some populations having developed neonicotinoid resistance (Luo et al. 2010; Rao et al. 2010; Venkataravanappa et al. 2013).

### Global distribution of *B. Tabaci* and its transmitted *begomoviruses*

**Fig. 2:**
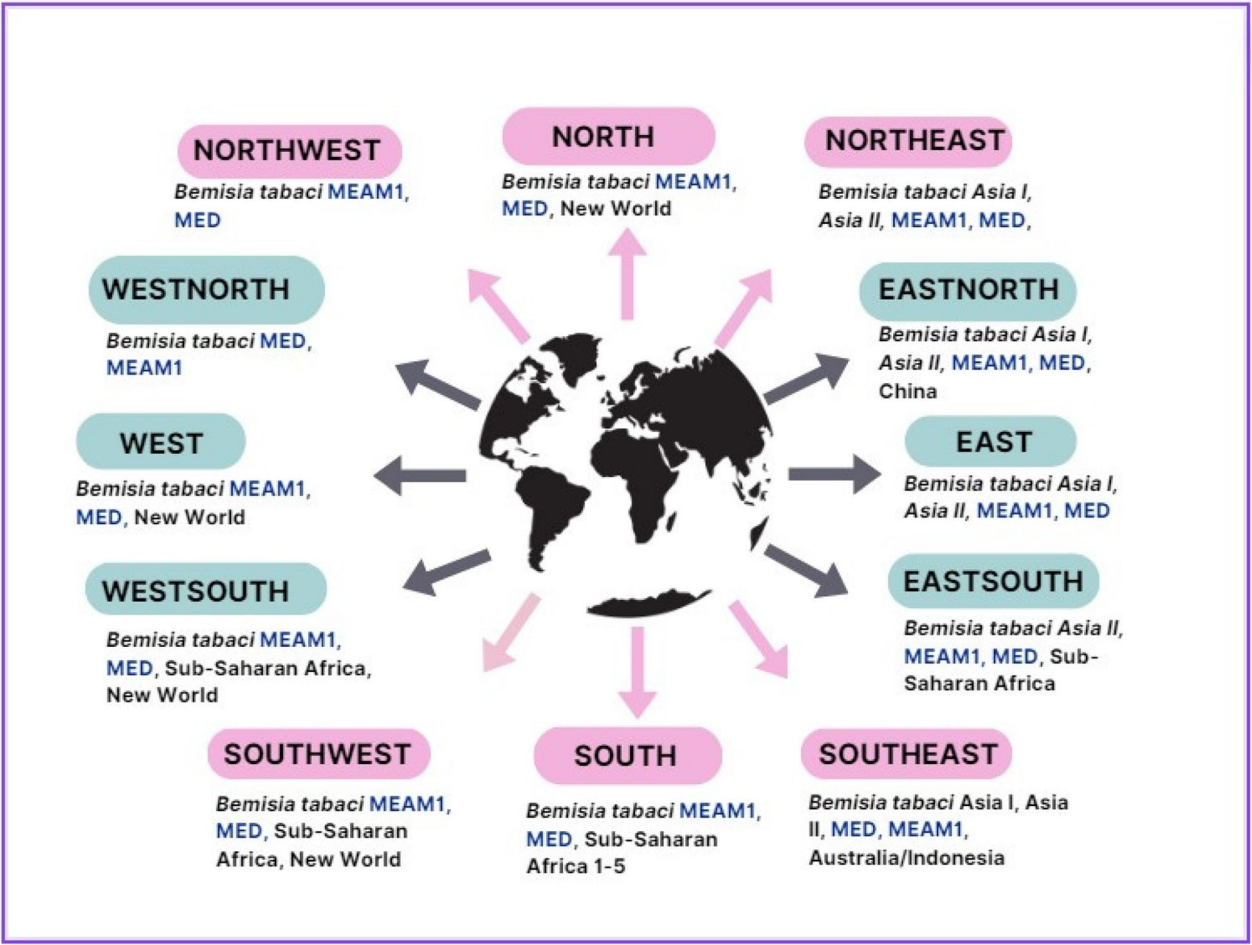
Geographical distribution of *B. tabaci* – showing dominant species present in global North, South, East, and West. Figure created with Canva

The spread of *begomovirus* is closely linked to *B. tabaci* vectors, especially *B. tabaci* MEAM1 and other cryptic species that transmit the virus more efficiently (Barman et al. 2022). These insects have spread to many parts of the world in recent decades, increasing the incidence and geographical distribution of *begomoviruses*. *Begomoviruses* such as OYVMV, are widespread across tropical and subtropical regions including India, Southeast Asia, the Middle East, Africa, and Southern Europe (Kanakala and Ghanim 2019), due to the spread of *B. tabaci* vectors. These are major constraints for okra production in India, Thailand, Egypt, Sudan, Saudi Arabia and Brazil among other countries globally. There are currently many recognized and studied cryptic species within the *B. tabaci* complex. Kanakala and Ghanim (2019) conducted a global analysis of *B. tabaci* distribution and diversity using available DNA sequences derived from the mitochondrial cytochrome c oxidase I gene (mtCOI sequences) from 82 countries. They identified two new possible species - Asia II 13 from India and Spain 1 from Morocco, in addition to the previously reported 42 species. The analysis revealed that Asia contains the highest diversity of *B. tabaci*, with 28 native and invasive species distributed across 13 countries. The Middle East Asia Minor I and II species (MEAM1 and MEAM2), were the most widespread globally. This confirmed the findings of an earlier study by De Barro et al. (2011). MEAM1 was reported in 42 countries across Asia, Africa, Europe, North and South America, while MED was found in 44 countries across these regions (Fig. 2). Other globally distributed species were Asia I, Asia II 1, Asia II 5–7, China 1–3, and Sub-Saharan Africa 1. Many species appeared restricted to single countries like Asia I-India in India, Asia II 2 in China, and Sub-Saharan Africa 5 in Uganda (Sseruwagi et al. 2005). Overall, the results provide an updated framework on the global diversity and distribution of this agriculturally important pest species complex (Kanakala and Ghanim 2019).

### Life cycle and behavior of *B. Tabaci*

**Fig. 3:**
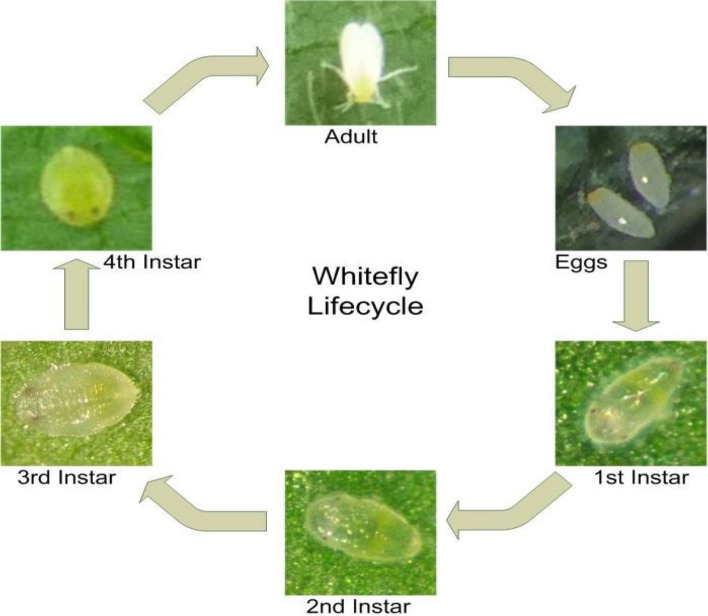
Life cycle of *B. tabaci*. Figure adapted from Barbedo 2014 with permission

*B. tabaci* goes through incomplete metamorphosis with egg, nymph, and adult life stages. (Fig. 3). Adults are small (0.8–1.2 mm) with a yellow body and white powdery wax coating on the wings (Li et al. 2021). Females lay oblong eggs on the undersides of leaves, attaching them vertically by a stalk. Egg-to-adult development takes approximately 3–5 weeks depending on temperature (Li et al. 2021). Four nymphal stages must be undergone before reaching the adult stage.

*B. tabaci* feeds primarily on phloem sap using its piercing-sucking mouthparts. Nymphs and adults like to feed on younger leaves, buds, and stems where nutrients are most plentiful. *B. tabaci* causes direct damage through phloem-feeding, inducing physiological disorders in the plant. However, the most significant impact is through the transmission of viruses during feeding. When viruliferous whiteflies feed, they can inoculate plants with viruses in as little as 5 min (Mehta et al. 1994). *B. tabaci* adults are active fliers and can disperse over long distances via wind currents. They exhibit a preference for younger, upper leaves for feeding and oviposition sites. These behaviors facilitate the rapid spread of viruses within and between crops. Management of *B. tabaci* is critical to prevent viral disease epidemics.

## Some *begomoviruses* associated with OYVMD

OYVMD is caused by a complex of *begomoviruses* belonging to the family Geminiviridae. This family has been classified into four genera: *Begomovirus*, *Mastrevirus*, *Curtovirus*, and *Topocuvirus* based on their host range, genome arrangement, and insect vectors. Of all these viruses, *begomoviruses* pose significant challenges to the cultivation of various dicotyledonous crops worldwide, making them one of the primary limiting factors in the production of these plants. The widespread impact of *begomoviruses* is evident from their global distribution, with 322 officially recognized species worldwide. India alone harbors about 82 of these species, which makes the Indian subcontinent a key hotspot for *begomovirus* diversity and likely origin. This extensive variety of *begomoviruses* contributes to their adaptability and persistence across different crops and regions (Malathi et al. 2017). *Begomoviruses* possess a genome composed of circular single-stranded DNA (ssDNA) molecules encapsidated in twinned icosahedral particles (Venkataravanappa et al. 2013b). The genome can be either monopartite, consisting of a single DNA component (DNA-A), or bipartite, consisting of two DNA components (DNA-A and DNA-B). In the nucleus, these ssDNA molecules are converted to double-stranded DNA (dsDNA) forms during replication (Mubeen et al. 2021). DNA-A encodes genes responsible for replication, encapsidation, suppression of host defenses, and control of gene expression (Kanakala and Ghanim 2019). In bipartite *begomoviruses*, DNA-B encodes proteins that facilitate intra- and intercellular movement within the plant host (Mubeen et al. 2021; Rojas et al. 2005).

Several *begomoviruses* have been reported to cause OYVMD in different regions worldwide, including OYVMV, Bhendi yellow vein mosaic virus (BYVMV), Okra enation leaf curl virus (OELCuV), Cotton leaf curl Multan virus (CLCuMuV), Okra yellow crinkle virus (OYCrV), Radish leaf curl virus (RaLCV), and Okra mosaic virus (OkMV).

OYVMV, one of the well-studied causes of OYVMD, has a monopartite genome of ~ 2.7 kb (Zhou et al. 1998). BYVMV, another monopartite *begomovirus*, has been associated with OYVMD in India (Jose and Usha 2003). In contrast, OELCuV, which causes OYVMD in India, has a bipartite genome (Venkataravanappa et al. 2013b). CLCuMuV, a *begomovirus* with a monopartite genome, has been reported to infect okra in Pakistan (Hameed et al. 2014). OYCrV, a bipartite *begomovirus*, has been associated with okra leaf curl disease in Mali, West Africa (Kon et al. 2009). RaLCV, a monopartite *begomovirus*, has been found to cause OYVMD in India (Pradhan and Mishra 2020), while OkMV, a *begomovirus* with a monopartite genome, has also been reported to infect okra in India (Sanwal et al. 2016).These *begomoviruses* share similarities in many ways, including their mode of acquisition, transmission and their genome structure.

### Genome organization and diversity among *begomoviruses* causing OYVMD

While the *begomoviruses* causing OYVMD share similarities in their genome structure, there are notable differences in some ways, including the number and arrangement of their open reading frames (ORFs) as well as their sequences (Venkataravanappa et al. 2013b) which influences the viruses’ host range, virulence, evolution, how efficiently each virus can be transmitted and their diagnosis and management since sequence information is crucial for developing accurate diagnostic tools and designing targeted management strategies, such as breeding for virus resistance in crops. For example, OYVMV, a monopartite *begomovirus*, encodes six proteins - Virion-sense strand, open reading frame (ORF) 1 (V1), Virion-sense strand, ORF 2 (V2), Complementary-sense strand, ORF 1 (C1), Complementary-sense strand, ORF 2 (C2), Complementary-sense strand, ORF 3 (C3), Complementary-sense strand, ORF 4 (C4) (Venkataravanappa et al. 2011; Ghosh et al. 2009; Jose and Usha 2003). However, this specific ORF arrangement may not be consistent across all *begomoviruses* causing OYVMD. For an instance, BYVMV, another monopartite *begomovirus*, has been reported to encode six ORFs similar to OYVMV (Jose and Usha 2003). In contrast, OELCuV, a bipartite *begomovirus*, may have a different ORF arrangement due to its split genome structure (Venkataravanappa et al. 2013b). CLCuMuV, a monopartite *begomovirus*, has been shown to encode six ORFs, including V1, V2, C1, C2, C3, and C4 (Hameed et al. 2014).

OYCrV, a bipartite *begomovirus*, also encodes six ORFs on its DNA-A component, including AV1 CP, AV2 (pre-coat protein), AC1 (Rep), AC2 (TrAP), AC3 (REn), and AC4 (symptom determinant) (Kon et al. 2009). However, the DNA-B component of OYCrV encodes two additional ORFs, BC1 and BV1, which are involved in virus movement (Kon et al. 2009). Additionally, RaLCV, a monopartite *begomovirus*, has been reported to encode six ORFs, similar to OYVMV (Pradhan and Mishra 2020), while OkMV, a monopartite *begomovirus*, encodes five ORFs, including V1, V2, C1, C2, and C3, but lacks the C4 ORF (Sanwal et al. 2016).

**Fig. 4:**
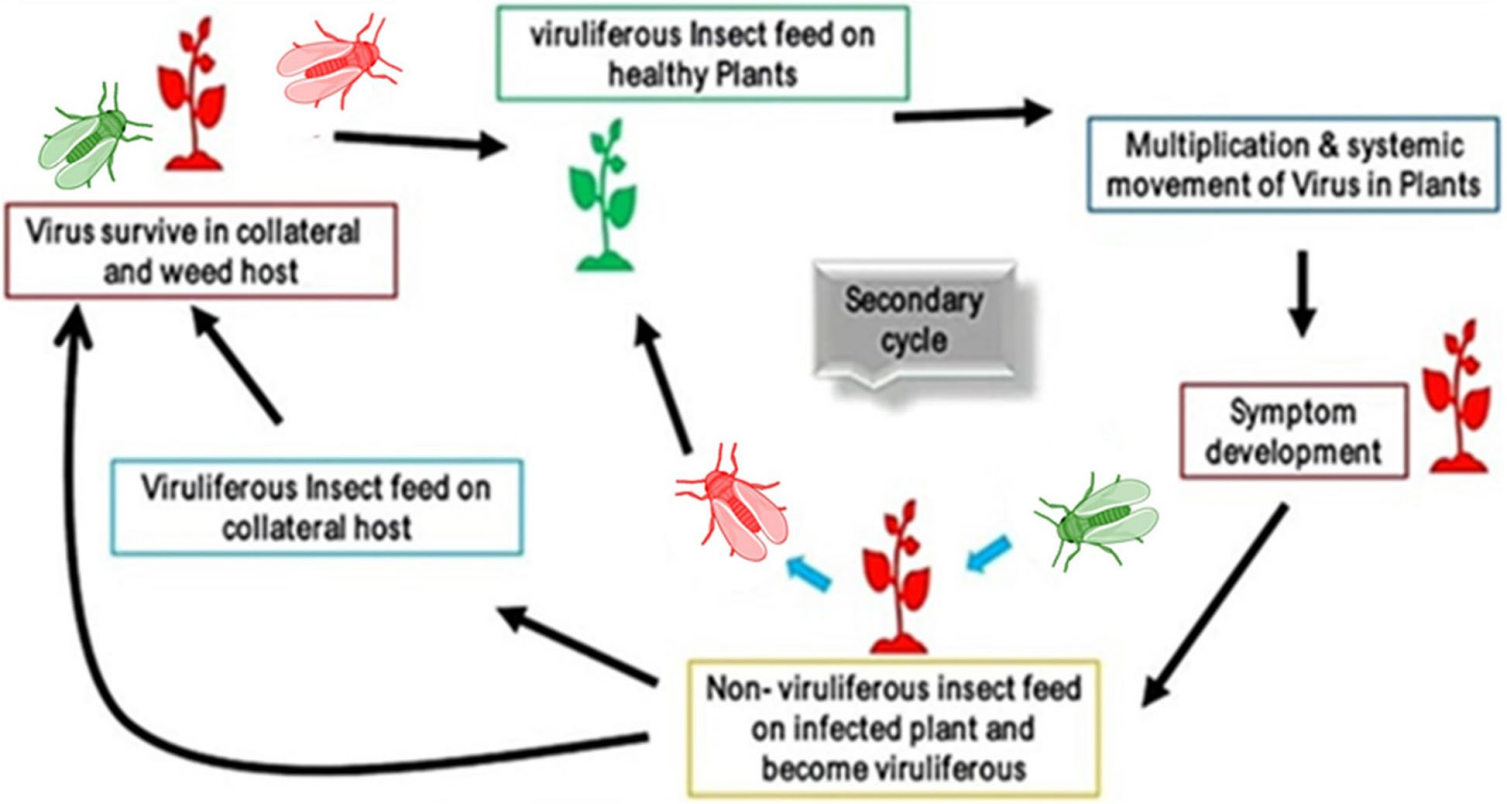
Disease cycle of okra yellow vein mosaic disease. This illustrates how *begomoviruses* are transmitted by *B. tabaci*. The cycle begins when *B. tabaci* feeds on a virus-infected okra plant, acquiring the virus along with the plant sap. The virus particles then circulate through the insect’s body, passing from the gut to the hemolymph and finally reaching the salivary glands. Once the virus has accumulated in the salivary glands, *B. tabaci* becomes capable of transmitting it to healthy okra plants during subsequent feeding. As *B. tabaci* probes and feeds on the phloem of a healthy plant, it introduces the virus particles through its saliva, initiating a new infection. The virus then replicates and spreads systemically throughout the okra plant, causing characteristic yellow vein mosaic symptoms. Figure created with BioRender.Com

These are evident that while *begomoviruses* causing OYVMD share a similar overall genome structure, there can be variations in the number and arrangement of their ORFs. Some *begomoviruses* may lack certain ORFs, such as C4 in the case of OkMV, while others, like OYCrV, may have additional ORFs encoded by their DNA-B component. These differences in genome organization contribute to the diversity among *begomoviruses* causing OYVMD and may have implications for their replication, pathogenicity, and host range. However, the transmission of the virus and the disease cycle entirely (Fig. 4) of the resulting OYVMD remains the same for all *begomoviruses*.

### Symptoms and impact on Okra plants

*Begomovirus* (such as BYVMV or OYVMV) infection causes distinctive yellow vein net symptoms on okra leaves. Initial symptoms appear as the clearing of veins which progress to a yellow vein net covering the entire leaf surface. Characteristic symptoms of virus-infected okra plants include chlorosis, dwarfing, and yellowing of veins and fruits (Mubeen et al. 2021). Plants infected at an early growth stage are severely stunted and experience extensive defoliation and yield loss. The fruit becomes small, hard, and twisted. Under favorable conditions for virus spread, *begomovirus* can cause total (100%) yield loss. The virus has a major impact on okra production in tropical and subtropical regions.

## *Begomovirus* acquisition and transmission mechanisms by *B. Tabaci*

**Fig. 5:**
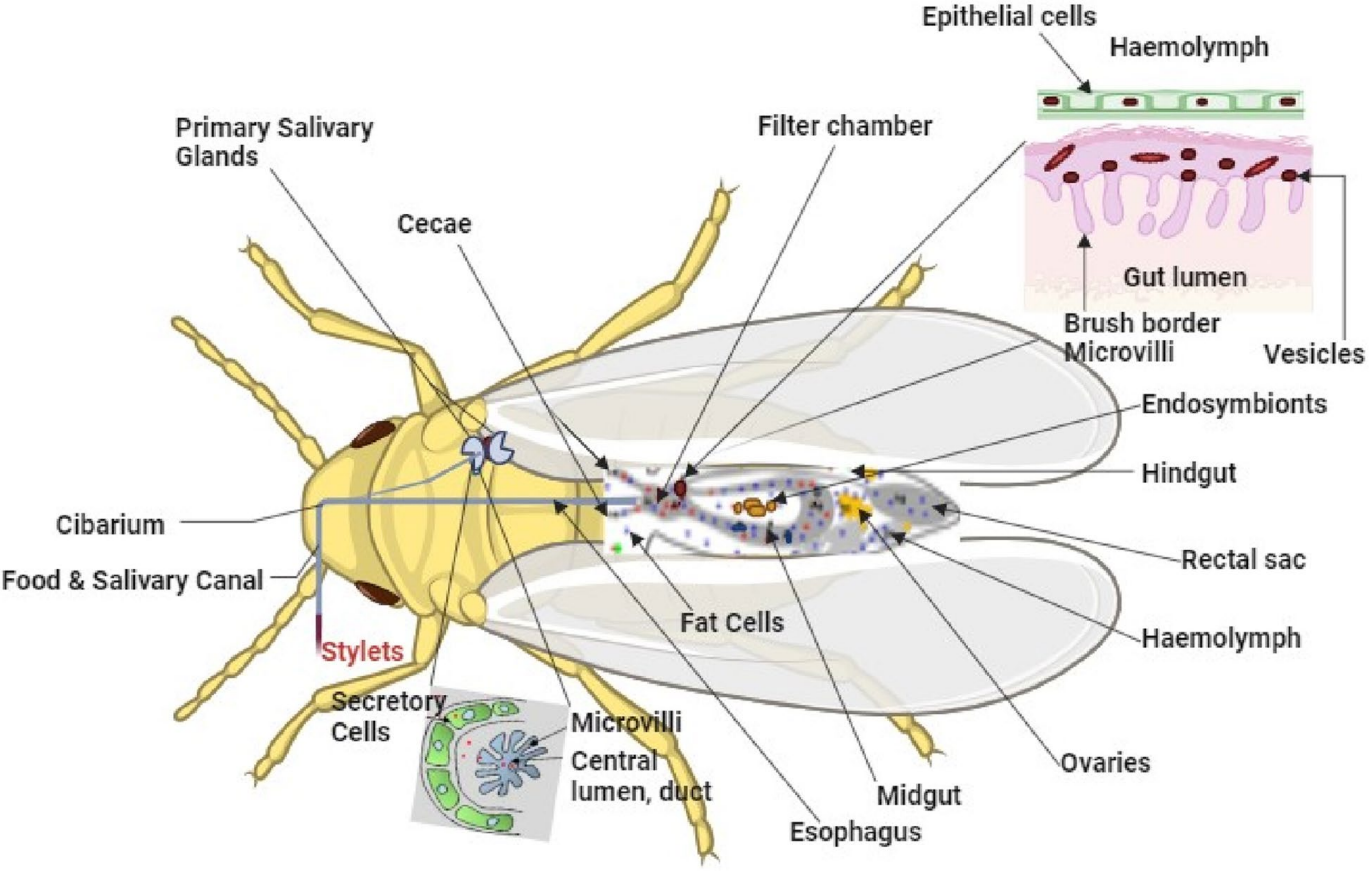
Schematic diagram showing organs and cells that are involved in *begomovirus* movement. The key virus movement locations in the midgut and primary salivary glands are highlighted and enlarged in the figure to clearly illustrate the detailed processes occurring within these insect tissues. Created with BioRender.com

The acquisition from infected host is done when *B. tabaci* feeds on virus-infected okra plants through some of its organs and cells. *B. tabaci* acquires virions into their stylets and foregut (Fig. 5). The major capsid protein V1 mediates binding to *B. tabaci* stylets, allowing *begomovirus* to be retained after ingestion (Horníková et al. 2016). Next is the virus movement into salivary glands. In this process virus particles cross gut epithelial barriers and pass into the hemolymph to reach the salivary glands. V1 again facilitates binding and entry into these glands. After this, the virus transmission via salivation begins. Once inside salivary glands, virus replication leads to accumulation and secretion of virus particles into saliva. When *B. tabaci* subsequently feeds on healthy plants, virus is egested into okra tissues. This begins the host infection in which inoculation into plant cells leads to viral replication, likely mediated by V1/V2 proteins which enable nuclear import and initiation of rolling circle replication of the viral genome (Venkataravanappa et al. 2011). These mechanisms of virus acquisition from infected host, movement into salivary glands, transmission via salivation, and new (uninfected) host infection are detailed in Sections Acquisition mechanisms and feeding behavior of B. tabaci, Begomovirus acquisition and factors influencing acquisition efficiency and Begomovirus transmission mechanisms and efficiency to healthy plants.

### Acquisition mechanisms and feeding behavior of *B. Tabaci*

*B. tabaci* feeds directly from the phloem sieve elements of plants using their piercing-sucking mouthparts (Jiang et al. 2000). Acquisition of viruses occurs during sustained phloem feeding. *B. tabaci* feeding behavior has been studied using the electrical penetration graph (EPG) technique, which shows a sequence of waveforms matching specific feeding activities (Jiang et al. 2000). Briefly, *B. tabaci* first penetrate leaf tissue with their stylets to reach the phloem, seen as potential drops in EPG recordings. They then engage in sustained passive phloem ingestion, represented by the waveform E2. This E2 phase is when acquisition of phloem-limited viruses occurs (Jiang et al. 2000). The duration of E2 correlates positively with virus acquisition, as longer phloem feeding allows increased viral uptake (Ning et al. 2015). It is important to note that while *B. tabaci* is feeding on virus-infected okra plants, thus ingesting the virus, it also ingests phloem sap (the feed) at the same time. Therefore, the virus particles are transported through the food canal into the cibarium and precibarium. As a circulative virus, *begomovirus* then moves from the cibarium into the esophagus, which carries it into the filter chamber and midgut. A major site of *begomovirus* accumulation is the microvilli of the midgut epithelial cells (Fig. 4), where the virus enters the cells through receptor-mediated endocytosis (Akad et al. 2004, 2007; Morin et al. 1999).

### *Begomovirus* acquisition and factors influencing acquisition efficiency

During sustained E2 phloem-feeding, *B. tabaci* ingest virions along with plant sap. Specific molecular interactions likely mediate viral particle binding to *B. tabaci* stylets and foregut lining, but these are still unclear for some *begomoviruses*. For other *begomoviruses*, the CP is necessary for insect transmission, suggesting it is involved in vector binding (Liu et al. 2013). Acquisition rates for virus increase with longer access periods on infected plants and reach a maximum after 24–48 h. Higher virus titer in the plant also increases *B. tabaci* acquisition.

During acquisition, *B. tabaci* ingests viral DNA-A and DNA-B particles (only DNA-A for monopartite *begomoviruses*) while feeding on okra phloem infected with both genomic components. Virions rapidly reach the cibarium via the food canal within 10 min of initiating ingestion. After 40 min, virus accumulates in the esophagus and filter chamber region of the midgut. Clathrin-mediated endocytosis enables the virus to cross the midgut epithelium into vesicles. Virus particles traverse midgut cell cytoplasm and exit basally between 90 and 120 min post-acquisition, entering the haemolymph. Here, GroEL proteins released from *B. tabaci* endosymbionts sequester and protect virus particles. Association with GroEL allows the virus to survive degradation by haemolymph components during circulation. Within 4–7 h, the virus penetrates the secretory cells of the primary salivary glands, a process facilitated by capsid protein interactions. Following a latent period (where applicable) of 8 + hours, *B. tabaci* can transmit virus to healthy okra plants during phloem feeding. Inoculation occurs rapidly, with virus detectable in recipient plants within 30 min of ingestion. Continuous acquisition feeding on infected okra for 12–48 h allows virus to accumulate to a threshold level in *B. tabaci*, plateauing around 600 million DNA-A and DNA-B genomes per insect. The bulk of viral particles are harbored in the midgut and filter chamber rather than the salivary glands. *Begomovirus* can persist at transmissible levels in *B. tabaci* for life following acquisition. However, as vectors age and degrade the virus over time, transmission efficiency slowly declines. For bipartite *begomoviruses*, both DNA-A and DNA-B are required for successful infection of plants, so transmission depends on adequate acquisition and preservation of both components. The persistent circulative transmission cycle of the virus perpetuates YVMD in okra crops. As long as viruliferous *B. tabaci* can feed on healthy okra plants, they will continue inoculating the plants with virus and inducing viral infection and OYVMD.

However, many biotic and abiotic factors can affect the efficiency of *begomovirus* acquisition by *B. tabaci*. For example, plant nitrogen status alters amino acid composition in the phloem sap, which affects *B. tabaci* feeding behavior and virus acquisition (Mubeen et al. 2021). Higher temperature decreases E2 phloem ingestion, also reducing virus acquisition. *B. tabaci* sex and biotype influence feeding behavior and acquisition capability, with B biotype more efficient than A (Lu et al. 2017). Plant resistance factors that alter phloem accessibility or feeding behavior also reduce *B. tabaci* acquisition of virus.

### *Begomovirus* transmission mechanisms and efficiency to healthy plants

After acquisition, *begomovirus* is retained within the *B. tabaci* vector as it circulates through the hemolymph and eventually reaches the salivary glands (Ghanim 2014). Upon reaching the primary salivary glands, the virus accumulates in specific cells near the central secretory region. This process is critical for transmission specificity and subsequent inoculation. When *B. tabaci* feeds on uninfected okra plants, the virus is egested along with saliva into the phloem, leading to infection and characteristic yellow vein mosaic symptoms within 2–3 weeks (Czosnek et al. 2017).

Therefore, it is well understood that transmission to new host plants occurs via salivation during phloem feeding. However, using EPG, two transmission-associated waveforms have been identified in this process: E1 represents salivary secretion into the phloem, while E2 correlates with passive phloem ingestion (Jiang et al. 2000). The E1 waveform is critical for inoculation of *begomoviruses* like OYVMV during brief salivary secretions (Jiang et al. 2000). Transmission efficiency is positively correlated with total E1 duration, indicating this waveform’s importance in viral inoculation. Researching further and understanding this feeding behavior that is linked to the transmission of *begomoviruses* such as OYVMV can inform management targeting the vector.

Generally, *begomovirus* transmission follows a persistent-circulative model, requiring a latent period between acquisition and transmission (Barman et al. 2022). This is applicable to most *begomoviruses* causing OYVMD except for a few such as the TYLCV which can be transmitted in a persistent-circulative manner without requiring a latent period (Bragard et al. 2013). Latency periods over 48 h have been reported for some *begomoviruses* (such as OYVMV), during which time the virus moves through the vector before reaching the salivary glands. Upon subsequent feeding, virions are inoculated into plant tissues via *B. tabaci* saliva during the E1 salivation waveform (Stafford et al. 2011). Virus transmission rates increase with longer feeding periods, plateauing after 48–72 h. Higher virus titer in the vector also increases transmission efficiency. Reducing virus titer in *B. tabaci* could potentially decrease circulative transmission to new host plants.

Venkataravanappa et al. (2017) conducted several experiments to compare the transmission efficiency of a *begomovirus* (OYVMV) by two cryptic species of *B. tabaci*, MEAM1 and Asia I. They found that MEAM1 transmitted OYVMV more efficiently than Asia I, requiring fewer insects per plant, shorter acquisition access and inoculation access periods, and ability to transmit to older host plants. A single MEAM1 adult could transmit OYVMV at 10% efficiency, while Asia I required 2–3 whiteflies for 50% transmission. The minimum acquisition access period was 15 min for MEAM1 and 20 min for Asia I to successfully transmit OYVMV. Similarly, minimum inoculation access period was 15 min for MEAM1 and 20 min for Asia I. Both species attained 100% transmission with inoculation access of 12 h or longer. MEAM1 maintained 100% transmission even on 2-week-old okra plants, while Asia I efficiency dropped on older seedlings. Overall, the results clearly demonstrate the superior vector competence of MEAM1 over Asia I for transmitting the virus in okra.

## Molecular interactions between *B. Tabaci*, *Begomovirus*, and okra plants

### *B. tabaci* -mediated virus entry, replication, and movement into plants

*B. tabaci* as a phloem-feeding insect can transmit numerous plant viruses in a persistent-circulative manner during feeding. When *B. tabaci* feeds on virus-infected plants, they ingest virions along with phloem sap. The *begomoviruses* are able to pass through the gut lumen into the hemocoel and ultimately reach the salivary glands, where they can be inoculated into new (uninfected) host plants (Ghanim 2014). Specific molecular interactions between viral proteins and *B. tabaci* vectors facilitate this circulative transmission. For example, the CP of tomato yellow leaf curl virus (TYLCV), a *begomovirus*, has been shown to mediate binding to *B. tabaci* guts (Liu et al. 2013).

After transmission into plant tissues by *B. tabaci*, the virus enters okra plant cells, disassembles and replicates their genomes, then spreads systemically through the plant. It is during this interaction that the ssDNA genomes of *begomovirus* is released into the nucleus where it is converted to dsDNA and replicates using the host replication machinery (Venkataravanappa et al. 2013b). Viral proteins mediate cell-to-cell movement through plasmodesmata and long-distance transport via the phloem. Viruses often induce morphological changes in plasmodesmata to facilitate intercellular movement. *Begomoviruses* encode proteins that facilitate their movement within the host plant. In bipartite *begomoviruses*, the DNA-B component encodes a movement protein (MP) that is involved in cell-to-cell movement through plasmodesmata, and in some cases, this MP has been shown to increase the plasmodesmatal size exclusion limit. In monopartite *begomoviruses*, the DNA-A component encodes proteins, such as the CP and/or C4 protein, which have been implicated in cell-to-cell movement, although their specific functions may vary among different species.

### Plant defense responses and virus evasion strategies

Plants have evolved sophisticated multi-layered defense mechanisms against viral pathogens, including *begomoviruses* that cause OYVMD. The first line of defense is often RNA silencing, a sequence-specific antiviral mechanism (Pumplin and Voinnet 2013). When *begomoviruses* replicate, they produce double-stranded RNA intermediates that are recognized by plant Dicer-like (DCL) enzymes. These DCLs cleave the viral dsRNA into small interfering RNAs (siRNAs) of 21–24 nucleotides in length (Blevins et al. 2006). The siRNAs are then loaded onto Argonaute (AGO) proteins, forming RNA-induced silencing complexes (RISC) that target and cleave complementary viral RNA sequences, effectively limiting viral replication and spread (Musidlak et al. 2017; Wang et al. 2011).

However, *begomoviruses* causing OYVMD have evolved counter-defense strategies to overcome RNA silencing. Many encode RNA silencing suppressors (RSSs) that interfere with various steps of the silencing pathway. For instance, the AC2 protein of BYVMV has been shown to suppress both transcriptional and post-transcriptional gene silencing by interacting with and inactivating adenosine kinase (ADK), an enzyme involved in the methyl cycle (Buchmann et al. 2009). Similarly, the βC1 protein encoded by the DNA-β satellite associated with BYVMV can suppress RNA silencing by interacting with and inactivating the plant S-adenosyl methionine decarboxylase 1 (SAMDC1) enzyme, which is involved in the methylation-dependent antiviral defense pathway (Kumar et al. 2015).

In addition to RNA silencing, plants employ hormone-mediated defenses against viral infections. Salicylic acid (SA) plays a crucial role in activating systemic acquired resistance (SAR) against viruses. Upon infection, plants increase SA production, leading to the expression of pathogenesis-related (PR) genes and the establishment of a broad-spectrum resistance (Carr et al. 2010; Musidlak et al. 2017). However, some *begomoviruses* causing OYVMD have evolved mechanisms to manipulate these hormone signaling pathways. For example, the C4 protein of (OELCuV), a Geminivirus, has been found to interfere with the brassinosteroid signaling pathway, thereby altering plant development and potentially suppressing defense responses (Mei et al. 2020).

Plant innate immunity also contributes to antiviral defense through the recognition of pathogen-associated molecular patterns (PAMPs) by pattern recognition receptors (PRRs). This recognition triggers PAMP-triggered immunity (PTI), characterized by the production of reactive oxygen species, callose deposition, and the activation of defense-related genes (Nicaise 2014). Some plants have evolved resistance (R) genes that recognize specific viral effectors, leading to effector-triggered immunity (ETI) and often resulting in a hypersensitive response (HR) with localized cell death to restrict viral spread (Mandadi and Scholthof 2013).

*Begomoviruses* causing OYVMD, in turn, have developed strategies to evade or suppress these immune responses. The AC2 protein of BYVMV has been shown to interact with and inhibit the function of SNF1-related kinase (SnRK1), a key regulator of plant metabolism and stress responses (Shen et al. 2011; Shen et al. 2009). Moreover, some *begomoviruses* can exploit the plant’s own regulatory mechanisms to their advantage. The Rep protein of a *begomovirus* interacts with and alters the function of plant proliferating cell nuclear antigen (PCNA), affecting DNA replication and repair processes, which may indirectly impact host defense responses (Bagewadi et al. 2004).

Deep and accurate scientific knowledge about these complex molecular interactions between *begomoviruses* causing OYVMD and plant defense mechanisms is important for developing effective strategies to manage the disease. Therefore, future research should focuse mainly on enhancing plant natural defense responses or disrupting viral counter-defense mechanisms since it could lead to novel and sustainable approaches for controlling *begomovirus* infections in okra.

### Host-Pathogen-Vector dynamics: a missing piece in *Begomovirus* ecology

The transmission and epidemiology of *begomoviruses* involve complex molecular interplay between the *B. tabaci* vector, the *begomovirus* pathogen, and susceptible okra plants. Successful circulative transmission relies on specific binding between virus CP in some cases and vector recognition factors to enable virion uptake, circulation, and delivery into host plants (Ghanim 2014). For example, the major capsid protein (V1) mediates interactions with *B. tabaci* stylets and foregut lining to facilitate viral retention after acquisition (Horníková et al. 2016).

Recent studies have revealed intricate details of these interactions in the okra-*begomovirus*-whitefly system. The CP of BYVMV has been shown to interact with the midgut protein Hsp16 in *B. tabaci*, which is crucial for virus transmission (Ohnesorge and Bejarano 2009). This interaction is thought to protect the virus from degradation during its passage through the insect vector.

Within the vector, virus movement across midgut and salivary tissues is enabled by molecular compatibilities with insect proteins like Hsp70, CypB, and PGRP (Czosnek et al. 2017). Additionally, the GroEL chaperonin produced by *B. tabaci* endosymbionts has been found to interact with the CP of BYVMV, leading to the facilitation of virus circulation in the hemolymph (Götz et al. 2012).

Upon reaching the salivary glands and accumulation to transmissible titers, the virus can be egested into plant tissue during subsequent feeding. Viral particles likely interact with okra cell surface receptors to penetrate tissues. Recent research has identified a potential receptor for viruses, a plasma membrane-localized leucine-rich repeat receptor-like kinase (LRR-RLK), which interacts with the viral CP and may facilitate virus entry into plant cells (Dievart et al. 2020; Soltabayeva et al. 2022). Inside plant cells, *begomovirus* genes direct replication, intra- and intercellular spread through plasmodesmata, and systemic movement through vascular tissues (Jose and Usha 2003). The Rep protein of BYVMV has been shown to interact with plant retinoblastoma-related protein (RBR), altering the cell cycle to create a favorable environment for virus replication (Chandran et al. 2014).

However, okra has intrinsic antiviral Ribonucleic Acid interference (RNAi) machinery that processes viral RNA into small interfering RNA (siRNAs) that target complementary sequences for degradation. *Begomoviruses* such as OYVMV encode silencing suppressor proteins like V2 to subvert these plant defenses (Venkataravanappa et al. 2011). The AC4 protein of BYVMV has been identified as a potent suppressor of post-transcriptional gene silencing, interfering with the plant’s RNAi-based antiviral response (Amin et al. 2011). The molecular factors mediating replication and spread in the host, evasion of plant immune responses, plus specific capsid-vector-host protein binding compatibilities underlying circulative transmission together facilitate virus infection, yellow vein mosaic symptoms, and epidemic spread. Interestingly, recent studies have shown that BYVMV infection alters the metabolic profile of okra plants, making them more attractive to *B. tabaci* and potentially enhancing virus spread (Malathi et al. 2017).

Proteomics research can provide insights into interrupting these tripartite interactions. For instance, a comparative proteomic analysis of resistant and susceptible okra varieties infected with BYVMV revealed differential expression of proteins involved in photosynthesis, stress response, and metabolism, providing potential targets for enhancing okra resistance to the virus (Venkataravanappa et al. 2018). Thorough study assessing these complex interactions is important for developing effective management strategies for OYVMD and increasing okra production. Future research on okra-*begomovirus*-*B. tabaci* relationship should focus on simplifying and making clear the molecular mechanisms of virus acquisition, transmission, and plant infection, as well as identifying key molecular factors in the plant’s defense response that could be enhanced to improve resistance against *begomoviruses*.

## Impact of environmental factors on *Begomovirus* transmission

### Temperature, humidity, and host plant characteristics

While investigating biotic stresses, researchers may inadvertently overlook the impacts of abiotic variables and additional external factors that influence disease progression or management. This oversight frequently occurs in studies of OYVMD and vector-mediated transmission specifically. This study consolidates the current understanding of several key abiotic, host-derived, and interaction-mediated determinants modulating *begomovirus* acquisition, circulation, inoculation, and epidemiology. Critically evaluating contributions from climatic conditions, crop nutritional status, endogenous metabolites, and community associations provides a more holistic platform to advance integrated disease mitigation practices. Explicitly integrating these external environmental impacts with improved knowledge of vector-virus compatibilities can strengthen approaches to managing this damaging okra pathogen. Table 1 summarizes key external environmental factors affecting *begomovirus* transmission with a detailed table caption.

**Table 1:** Some abiotic factors like temperature and humidity as well as host plant characteristics and interactions with other pathogens that affect *begomovirus* transmission. Abiotic factors like temperature and humidity can significantly influence virus transmission by *B. Tabaci*. Higher temperatures (30–35 °C) accelerate virus replication in plants and circulation through *B. tabaci* vectors, reducing latency periods (where applicable) and increasing transmission efficiency. However, extremely high temperatures above 35 °C negatively impact *B. tabaci* survival and transmission capability. Low relative humidity also impedes viral transmission, likely by altering *B. tabaci* behavior and longevity. Climate change models predict increasing temperatures and drought stress in many agricultural regions, which could potentially alter *begomovirus* epidemiology in complex ways. Host plant factors like nutrient status, secondary metabolites, and physical defenses can affect *B. tabaci* feeding and virus transmission. For example, okra cultivars with increased leaf trichome density or polyphenols see reduced *B. Tabaci* infestation and lower viral transmission rates. On the other hand, nitrogen deficiency decreases tomatine glycoalkaloids in tomato, increasing *B. tabaci* feeding time and virus transmission. Breeding okra cultivars with physical and chemical resistance traits could suppress vector behavior and virus spread. All of these are indicated in this table

Environmental Factor	Effect on Virus Transmission	References
Higher temperature (30–35 °C)	Accelerates virus replication and movement in vector; reduces latency period; increases transmission efficiency	Patil and Kimar, 1992
Extremely high temperature (> 35 °C)	Negatively impacts vector survival and capability	Xu et al. 2010
Low relative humidity	Impedes viral transmission, likely by altering vector behavior and longevity	Tsai and Wang 1996
Climate change (increasing temperature, drought stress)	Potential to alter *begomovirus* epidemiology in complex ways	Xu et al. 2010
Host plant nutrients (nitrogen status)	Affects amino acid composition in the phloem, altering feeding behavior and virus acquisition	Nawaz-ul-Rehman et al. 2010
Host secondary metabolites (leaf trichomes, polyphenols)	Reduce vector feeding and virus transmission in resistant varieties	Davis 2022b
Mixed infections with other pathogens	Can increase viral titers and transmission	Nawaz-ul-Rehman et al. 2010
Fungal endophytes	Induce systemic resistance against *begomovirus* and reduce vector populations	Nawaz-ul-Rehman et al. 2010

### Interactions with other pathogens

Mixed infections of *begomoviruses* (e.g., OYVMV with other *begomoviruses*) or satellites can influence disease synergisms and viral titers in plants, and can increase acquisition and transmission by *B. tabaci* (Nawaz-ul-Rehman et al. 2010). In the context of OYVMD, several studies have reported complex interactions between multiple *begomoviruses* and their associated satellites (Singh et al. 2021). For instance, Venkataravanappa et al. (2015) found that co-infection of okra plants with BYVMV and OELCuV resulted in more severe symptoms and higher viral titers compared to single infections. This synergistic interaction was attributed to the complementary functions of viral proteins from both viruses, which enhance their ability to suppress host defenses and promote virus replication. Similarly, the presence of betasatellites associated with *begomoviruses* can significantly impact disease severity and vector transmission. Kumar et al. (2014) demonstrated that the βC1 protein encoded by the Cotton leaf curl Multan betasatellite (CLCuMB) associated with BYVMV enhanced viral DNA accumulation and symptom severity in okra. This protein was found to suppress both transcriptional and post-transcriptional gene silencing mechanisms in the host plant. Thus, creating a more favorable environment for virus replication and spread.

*B. tabaci* co-infected with plant and insect viruses demonstrate altered behavior and enhanced *begomovirus* transmission. Jiu et al. 2007 reported that *B. tabaci* infected with Tomato yellow leaf curl China virus (TYLCCNV) showed increased fecundity and longevity, which potentially led to higher virus transmission rates. In the case of OYVMD, similar effects have been observed with BYVMV-infected *B. tabaci*, which exhibited enhanced performance on okra plants (Malathi et al. 2017). Furthermore, fungal endophytes in plants can induce systemic resistance against *begomovirus* and reduce *B. tabaci* populations (Sani et al. 2020). While not specifically studied in okra, research on tomato plants has shown that endophytic colonization by Beauveria bassiana can provide protection against whitefly (Qayyum et al. 2015). This suggests that similar endophyte-mediated resistance might be applicable to okra, potentially increasing resistance to *begomovirus* infection and reducing *B. tabaci* population and infestation. The mechanism of such resistance often involves upregulation of defense-related genes and production of secondary metabolites that deter insect feeding (Sani et al. 2020). Recent studies have also highlighted the role of plant viruses in modulating vector behavior to enhance their own transmission. Shi et al. (2018) demonstrated that TYLCCNV infection altered the composition of volatile organic compounds emitted by tomato plants, making them more attractive to *B. tabaci*. A similar phenomenon has been reported in which *begomovirus* infected plants were found to be more attractive to *B. tabaci* compared to healthy plants (Maluta et al. 2014). An in-depth knowledge about these complex interactions between multiple pathogens, vectors, and host plants will play a vital role in developing effective management strategies for OYVMD. Hence, future research should focus on understanding the molecular mechanisms underlying these interactions and exploring potential approaches to disrupt them, such as the use of beneficial endophytes or the development of okra varieties with broad-spectrum resistance to multiple *begomoviruses* and their associated satellites.

## Control strategies for *Begomovirus* transmission and OYVMD Management

According to Mubeen et al. 2021; there are several key strategies for managing OYVMD, including developing genetically resistant cultivars, controlling the *B. tabaci* vector, and using plant defense activators. Since multiple *begomoviruses* can cause OYVMD, developing okra cultivars with broad-spectrum *begomovirus* resistance is an important goal. Combining multiple resistance genes targeting different virus species or strains is a promising approach.

Screening okra genotypes has identified several with resistance or tolerance to some *begomoviruses* (like OYVMV), although no highly resistant cultivars are yet available. Both dominant and additive gene effects contribute to resistance. Molecular breeding approaches like gene pyramiding show promise for incorporating resistance. The *B. tabaci* vector can be managed by optimizing sowing dates, intercropping, weed removal, and applying chemical and botanical insecticides. Synthetic chemicals effectively reduce *B. tabaci* populations but have toxicity concerns. Extracts from plants like neem, tobacco, and chaste tree can control *B. tabaci* with less environmental impact (Davis 2022a). Exogenous application of defense signaling molecules such as salicylic acid and potassium phosphate activates okra’s endogenous resistance mechanisms. This induced systemic resistance inhibits the virus multiplication and protects against *B. tabaci* infestation. Integrating chemical elicitors into pest management programs reduces disease incidence.

Nutrient status also impacts OYVMD development. Nitrogen promotes crop growth but can increase *B. tabaci* populations. Maintaining optimal levels of minerals like potassium is important for photosynthesis and growth in infected plants. Secondary metabolites like phenols may contribute to viral resistance. An integrated disease management approach using resistant varieties, vector control, plant activators, and cultivation practices is needed to effectively minimize the impact of OYVMD on okra yield and quality. Continued breeding and biotechnological efforts will improve genetic resistance in this crop. Additionally, other practices such as cultural, biological, and chemical can also be used.

### Cultural practices and crop management

Several cultural and crop management practices can help suppress *B. tabaci* populations and virus transmission. Adjusting planting dates to avoid periods of peak *B. tabaci* infestation reduces virus inoculum sources. Intercropping okra with pest-repellent plants like coriander or marigold can deter *B. tabaci*. Optimizing fertilizer application rates improves plant vigor and resistance against viral infection and vectors. Removing infested crop residues and weeds eliminates alternate *B. tabaci* and virus reservoirs. While individual effects may be small, integrating multiple tactics will effectively minimize the virus transmission.

### Biological control agents

Various biocontrol agents have shown promise in controlling *B. tabaci* and reducing virus spread. Parasitoid wasps like Eretmocerus spp. can parasitize *B. tabaci* nymphs, while entomopathogenic fungi such as *Beauveria bassiana* infect adult insects (Qayyum et al. 2015). Entomopathogenic viruses, such as nucleopolyhedroviruses isolated from *B. tabaci*, could also be explored as an environmentally friendly *B. tabaci* management tool to reduce OYVMD spread. The fungi also induce plant resistance mechanisms against viruses. Predatory lady beetles and lacewings that feed on *B. tabaci* have also been evaluated. Implementing conservation biological control in cropping systems will suppress vector populations and virus transmission.

### Chemical control options

While insecticides are not directly active against plant viruses, controlling *B. tabaci* vectors with judicious insecticide use can reduce viral spread. Effective classes include neonicotinoids, pyrethroids, organophosphates, and insect growth regulators (Naranjo and Ellsworth 2009). However, overreliance on insecticides often leads to resistance development in *B. tabaci* populations. Using integrated management programs that combine minimal insecticide application with cultural tactics, biocontrol agents, and resistant cultivars is a more sustainable strategy to suppress vector-mediated viral transmission over the long term.

## Conclusion

In this paper, we have synthesized current knowledge on the transmission mechanisms of *begomoviruses* causing OYVMD by the vector *B. tabaci*, emphasizing the interactions between these viruses, their insect vector, and host plant. We have also identified gaps in understanding *begomovirus* - *B. tabaci* interactions and discussed future research directions that provide insights into interrupting *begomovirus* transmission, which is crucial for developing effective OYVMD management strategies. *B. tabaci* acquires *begomovirus* particles while feeding from the phloem of infected plants. Following a latent period (where applicable), virions circulate to the salivary glands and can be transmitted to new host plants during subsequent feeding. Molecular compatibilities between vector, virus, and host affect acquisition and transmission efficiency. Abiotic factors like temperature and humidity as well as host plant traits also influence vector behavior and virus epidemiology. A better understanding of these tripartite interactions can inform integrated management approaches. Further research is needed to further clarify the specific molecular mechanisms mediating *begomovirus* - *B. tabaci* interactions and quantify their effects on transmission efficiency under different environmental conditions. Detailed characterization of *B. tabaci* feeding behaviors associated with viral acquisition and inoculation using EPG could enhance targeted control methods. Additionally, identifying resistance genes in okra germplasm and breeding cultivars with traits that disrupt multiple vector-virus compatibilities represents a promising sustainable management strategy.

An integrated approach combining host plant resistance, cultural tactics to avoid peak vector populations, conservation of natural enemies, and minimal insecticide use is recommended to control virus transmission and OYVMD infection. Deploying resistant varieties expressing traits like increased leaf trichomes or secondary metabolites that deter *B. tabaci* can limit virus spread. Optimization of planting dates, intercropping, and fertilization also help suppress vectors and diseases. Conservation of biological control via natural enemies and biopesticides supplements other measures. Further research and optimization of integrated management programs are recommended since these will strengthen food security by mitigating yield losses from this damaging viral disease.

## Data Availability

Not applicable.

## Abbreviations

AbMV: Abutilon Mosaic Virus
ADK: Adenosine Kinase
AGO: Argonaute
BYVMV: Bhendi Yellow Vein Mosaic Virus
CLCuMB: Cotton Leaf Curl Multan Betasatellite
CLCuMuV: Cotton Leaf Curl Multan Virus
CP: Coat Protein
CypB: Cyclophilin B
DCL: Dicer-like
DNA: Deoxyribonucleic Acid
dsDNA: Double-Stranded DNA
EPG: Electrical Penetration Graph
ETI: Effector-Triggered Immunity
Hsp16: Heat Shock Protein 16
Hsp70: Heat Shock Protein 70
LRR-RLK: Leucine-Rich Repeat Receptor-Like Kinase
MEAM: Middle East-Asia Minor
MED: Mediterranean
MP: Movement Protein
mtCOI: Mitochondrial Cytochrome c Oxidase I
OELCuV: Okra Enation Leaf Curl Virus
OkMV: Okra Mosaic Virus
ORF: Open Reading Frame
OYCrV: Okra Yellow Crinkle Virus
OYVMD: Okra Yellow Vein Mosaic Disease
OYVMV: Okra Yellow Vein Mosaic Virus
PAMP: Pathogen-Associated Molecular Pattern
PCNA: Proliferating Cell Nuclear Antigen
PGRP: Peptidoglycan Recognition Protein
PR: Pathogenesis-Related
PRR: Pattern Recognition Receptor
PTI: PAMP-Triggered Immunity
RaLCV: Radish Leaf Curl Virus
RBR: Retinoblastoma-Related Protein
RISC: RNA-Induced Silencing Complex
RNAi: RNA Interference
RSS: RNA Silencing Suppressor
SA: Salicylic Acid
SAMDC1: S-Adenosyl Methionine Decarboxylase 1
SAR: Systemic Acquired Resistance
siRNA: Small Interfering RNA
SnRK1: SNF1-Related Kinase
ssDNA: Single-Stranded DNA
TYLCCNV: Tomato Yellow Leaf Curl China Virus
TYLCV: Tomato Yellow Leaf Curl Virus
YVMD: Yellow Vein Mosaic Disease
